# Supplementary material for: The carbon footprint of the U.S. multinationals’ foreign affiliates
Source: Nat Commun. 2019 Apr 11;10:1672. doi: 10.1038/s41467-019-09473-7 (PMC6459871; doi:10.1038/s41467-019-09473-7)
Supplement: Supplementary file 1 — Supplementary Information [file 41467_2019_9473_MOESM1_ESM.docx]

**The Carbon Footprint of the U.S. Multinationals’ Foreign Affiliates**

López, et al.

**SUPPLEMENTARY INFORMATION**

**Supplementary Figure 1: Countries covered by WIOD release 2013.**


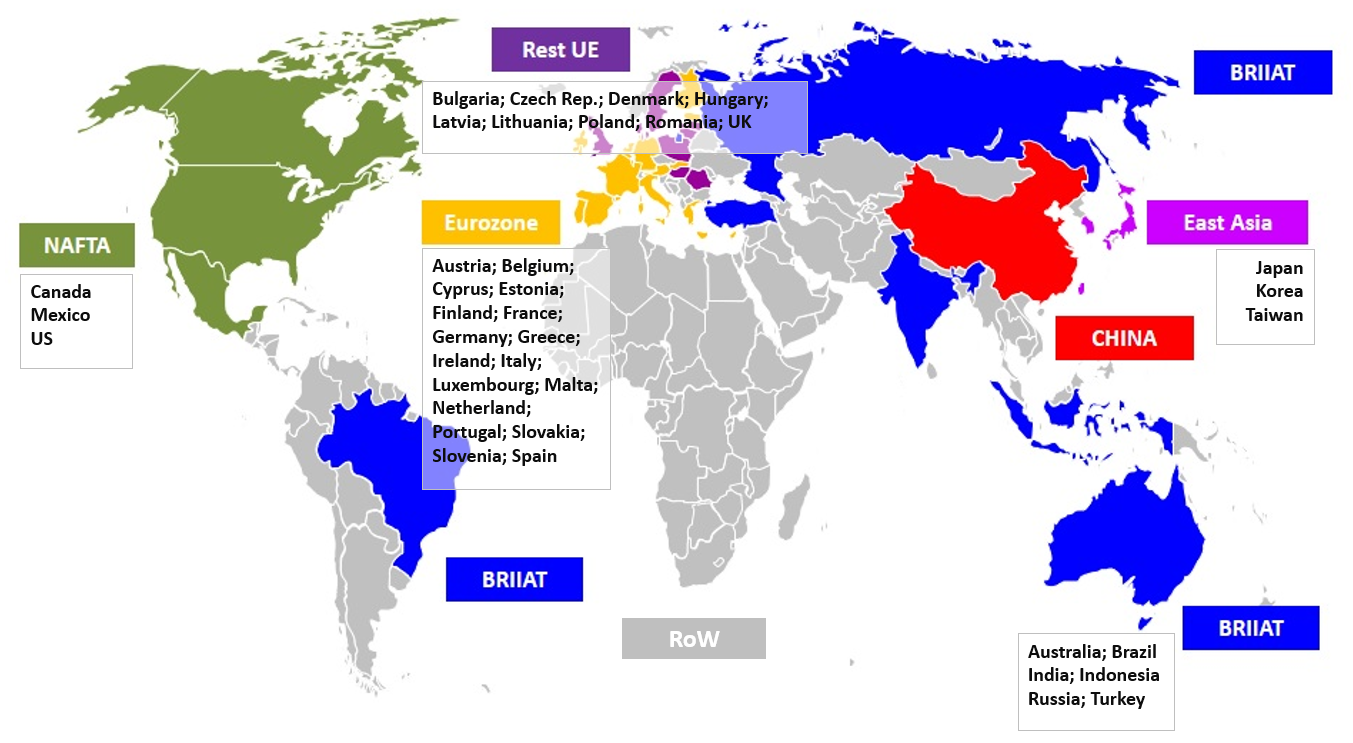


**Supplementary Table 1. US-MNE’ carbon footprint among emitter’s world rank (GtCO_2_)**

| **RANK** | **COUNTRY** | **PR** | **%** |  | **RANK** | **COUNTRY** | **PR** | **%** |
| --- | --- | --- | --- | --- | --- | --- | --- | --- |
| 1 | **CHN** | 8.0789 | 1590% |  | 22 | **NLD** | 0.1951 | 38% |
| 2 | **ROW** | 7.6189 | 1499% |  | 23 | **CZE** | 0.1167 | 23% |
| 3 | **U.S.** | 5.1767 | 1019% |  | 24 | **GRC** | 0.1084 | 21% |
| 4 | **IND** | 2.2556 | 444% |  | 25 | **BEL** | 0.1057 | 21% |
| 5 | **RUS** | 2.0296 | 399% |  | 26 | **ROU** | 0.1006 | 20% |
| 6 | **JPN** | 1.0193 | 201% |  | 27 | **DNK** | 0.0925 | 18% |
| 7 | **BRA** | 0.8267 | 163% |  | 28 | **PRT** | 0.0700 | 14% |
| 8 | **DEU** | 0.7475 | 147% |  | 29 | **FIN** | 0.0666 | 13% |
| 9 | **IDN** | 0.6067 | 119% |  | 30 | **SWE** | 0.0609 | 12% |
| 10 | **KOR** | 0.5906 | 116% |  | 31 | AUT | 0.0585 | 12% |
| 11 | **CAN** | 0.5785 | 114% |  | 32 | **HUN** | 0.0566 | 11% |
| **12** | **US-MNE** | **0.5082** | **100%** |  | 33 | **BGR** | 0.0535 | 11% |
| 13 | **GBR** | 0.5055 | 99% |  | 34 | **IRL** | 0.0451 | 9% |
| 14 | **AUS** | 0.5025 | 99% |  | 35 | **SVK** | 0.0385 | 8% |
| 15 | **MEX** | 0.4623 | 91% |  | 36 | **LTU** | 0.0206 | 4% |
| 16 | **ITA** | 0.3965 | 78% |  | 37 | **EST** | 0.0174 | 3% |
| 17 | **FRA** | 0.3665 | 72% |  | 38 | **SVN** | 0.0158 | 3% |
| 18 | **POL** | 0.3448 | 68% |  | 39 | **LVA** | 0.0107 | 2% |
| 19 | **TUR** | 0.3069 | 60% |  | 40 | **CYP** | 0.0086 | 2% |
| 20 | **TWN** | 0.3036 | 60% |  | 41 | **LUX** | 0.0032 | 1% |
| 21 | **ESP** | 0.2848 | 56% |  | 42 | **MLT** | 0.0027 | 1% |

**Supplementary Table 2. US-MNE´ producer carbon footprint (PF) by country (host country) (GtCO_2_)**

| **RANK** | **COUNTRY** | **MNE PF** | **%** |  | **RANK** | **COUNTRY** | **MNE PF** | **%** |
| --- | --- | --- | --- | --- | --- | --- | --- | --- |
| 1 | **ROW** | **0.1281** | 25.2% |  | 21 | **IDN** | **0.0041** | 0.8% |
| 2 | **CAN** | **0.0555** | 10.9% |  | 22 | **KOR** | **0.0038** | 0.8% |
| 3 | **GBR** | **0.0393** | 7.7% |  | 23 | **CZE** | **0.0029** | 0.6% |
| 4 | **DEU** | **0.0368** | 7.2% |  | 24 | **HUN** | **0.0029** | 0.6% |
| 5 | **CHN** | **0.0356** | 7.0% |  | 25 | **GRC** | **0.0022** | 0.4% |
| 6 | **BRA** | **0.0248** | 4.9% |  | 26 | **SWE** | **0.0019** | 0.4% |
| 7 | **IRL** | **0.0218** | 4.3% |  | 27 | **PRT** | **0.0017** | 0.3% |
| 8 | **FRA** | **0.0209** | 4.1% |  | 28 | **AUT** | **0.0015** | 0.3% |
| 9 | **AUS** | **0.0204** | 4.0% |  | 29 | **LUX** | **0.0010** | 0.2% |
| 10 | **MEX** | **0.0143** | 2.8% |  | 30 | **FIN** | **0.0008** | 0.2% |
| 11 | **IND** | **0.0133** | 2.6% |  | 31 | **DNK** | **0.0007** | 0.1% |
| 12 | **NLD** | **0.0131** | 2.6% |  | 32 | **BGR** | **---** | --- |
| 13 | **ITA** | **0.0117** | 2.3% |  | 33 | **CYP** | **---** | --- |
| 14 | **POL** | **0.0082** | 1.6% |  | 34 | **EST** | **---** | --- |
| 15 | **BEL** | **0.0081** | 1.6% |  | 35 | **LTU** | **---** | --- |
| 16 | **JPN** | **0.0081** | 1.6% |  | 36 | **LVA** | **---** | --- |
| 17 | **RUS** | **0.0079** | 1.6% |  | 37 | **MLT** | **---** | --- |
| 18 | **TUR** | **0.0062** | 1.2% |  | 38 | **ROU** | **---** | --- |
| 19 | **ESP** | **0.0057** | 1.1% |  | 39 | **SVK** | **---** | --- |
| 20 | **TWN** | **0.0049** | 1.0% |  | 40 | **SVN** | **---** | --- |

**Supplementary Table 3. Weighting US-MNE’ producer carbon footprint (host country) by each country producer responsibility (PR) (%)**

| **RANK** | **COUNTRY** | **US-MNE PF/PR** |  | **RANK** | **COUNTRY** | **US-MNE PF/PR** |
| --- | --- | --- | --- | --- | --- | --- |
| 1 | **IRL** | 48.38% |  | 22 | **ROW** | 1.68% |
| 2 | **LUX** | 31.48% |  | 23 | **TWN** | 1.60% |
| 3 | **CAN** | 9.59% |  | 24 | **FIN** | 1.19% |
| 4 | **GBR** | 7.78% |  | 25 | **JPN** | 0.79% |
| 5 | **BEL** | 7.71% |  | 26 | **DNK** | 0.73% |
| 6 | **NLD** | 6.70% |  | 27 | **IDN** | 0.68% |
| 7 | **FRA** | 5.72% |  | 28 | **KOR** | 0.65% |
| 8 | **HUN** | 5.05% |  | 29 | **IND** | 0.59% |
| 9 | **DEU** | 4.92% |  | 30 | **CHN** | 0.44% |
| 10 | **AUS** | 4.06% |  | 31 | **RUS** | 0.39% |
| 11 | **MEX** | 3.10% |  | 32 | **BGR** | --- |
| 12 | **SWE** | 3.07% |  | 33 | **CYP** | --- |
| 13 | **BRA** | 3.00% |  | 34 | **EST** | --- |
| 14 | **ITA** | 2.96% |  | 35 | **LTU** | --- |
| 15 | **AUT** | 2.64% |  | 36 | **LVA** | --- |
| 16 | **PRT** | 2.49% |  | 37 | **MLT** | --- |
| 17 | **CZE** | 2.45% |  | 38 | **ROU** | --- |
| 18 | **POL** | 2.39% |  | 39 | **SVK** | --- |
| 19 | **GRC** | 2.02% |  | 40 | **SVN** | --- |
| 20 | **TUR** | 2.01% |  | 41 | **U.S.** | --- |
| 21 | **ESP** | 1.99% |  |  |  |  |

**Supplementary Table 4. Consumer and host US-MNE’ carbon footprint (MNE CF, MNE PF) and MNE-US carbon balance by country (Kt CO_2_)**

| **COUNTRY** | **US-MNE PF (host country)** | **US-MNE PF (%)** | **US-MNE CF (consumer country)** | **US-MNE CF (%)** | **Difference consumer - host** |
| --- | --- | --- | --- | --- | --- |
| **U.S.** | 0 | 0.00% | 39616 | 7.80% | 39616 |
| **RUS** | 7928 | 1.56% | 12975 | 2.55% | 5047 |
| **JPN** | 8081 | 1.59% | 13330 | 2.62% | 5249 |
| **ITA** | 11731 | 2.31% | 15073 | 2.97% | 3343 |
| **ESP** | 5661 | 1.11% | 8529 | 1.68% | 2868 |
| **FRA** | 20949 | 4.12% | 23252 | 4.58% | 2303 |
| **TUR** | 6176 | 1.22% | 7961 | 1.57% | 1784 |
| **DEU** | 36767 | 7.23% | 38544 | 7.58% | 1777 |
| **GRC** | 2194 | 0.43% | 3450 | 0.68% | 1256 |
| **AUT** | 1545 | 0.30% | 2826 | 0.56% | 1281 |
| **IND** | 13313 | 2.62% | 14329 | 2.82% | 1017 |
| **SWE** | 1873 | 0.37% | 2883 | 0.57% | 1010 |
| **SVK** | 0 | 0.00% | 787 | 0.15% | 787 |
| **DNK** | 680 | 0.13% | 1413 | 0.28% | 734 |
| **AUS** | 20379 | 4.01% | 21088 | 4.15% | 709 |
| **ROU** | 0 | 0.00% | 664 | 0.13% | 664 |
| **PRT** | 1744 | 0.34% | 2366 | 0.47% | 622 |
| **FIN** | 793 | 0.16% | 1302 | 0.26% | 508 |
| **SVN** | 0 | 0.00% | 321 | 0.06% | 321 |
| **BGR** | 0 | 0.00% | 247 | 0.05% | 247 |
| **LTU** | 0 | 0.00% | 187 | 0.04% | 187 |
| **CYP** | 0 | 0.00% | 153 | 0.03% | 153 |
| **LVA** | 0 | 0.00% | 116 | 0.02% | 116 |
| **EST** | 0 | 0.00% | 97 | 0.02% | 97 |
| **MLT** | 0 | 0.00% | 77 | 0.02% | 77 |
| **LUX** | 1017 | 0.20% | 701 | 0.14% | -316 |
| **KOR** | 3813 | 0.75% | 3435 | 0.68% | -378 |
| **IDN** | 4102 | 0.81% | 3618 | 0.71% | -484 |
| **HUN** | 2855 | 0.56% | 1933 | 0.38% | -922 |
| **BRA** | 24814 | 4.88% | 23817 | 4.69% | -997 |
| **CZE** | 2862 | 0.56% | 1836 | 0.36% | -1026 |
| **POL** | 8227 | 1.62% | 6865 | 1.35% | -1362 |
| **TWN** | 4855 | 0.96% | 3465 | 0.68% | -1390 |
| **GBR** | 39312 | 7.74% | 36785 | 7.24% | -2527 |
| **BEL** | 8148 | 1.60% | 4763 | 0.94% | -3385 |
| **MEX** | 14350 | 2.82% | 10734 | 2.11% | -3616 |
| **CHN** | 35596 | 7.00% | 31139 | 6.13% | -4457 |
| **NLD** | 13065 | 2.57% | 7655 | 1.51% | -5411 |
| **IRL** | 21819 | 4.29% | 15687 | 3.09% | -6132 |
| **CAN** | 55475 | 10.92% | 47820 | 9.41% | -7655 |
| **ROW** | 128094 | 25.20% | 96378 | 18.96% | -31716 |

**Supplementary Table 5. Weighting US-MNE’ carbon footprint (MNE CF) by each country consumer responsibility (CR) (%)**

| **RANK** | **COUNTRY** | **US-MNE CF/CR** |  | **RANK** | **COUNTRY** | **US-MNE CF/CR** |
| --- | --- | --- | --- | --- | --- | --- |
| 1 | **IRL** | 26.36% |  | 22 | **SVK** | 1.87% |
| 2 | **LUX** | 10.45% |  | 23 | **CZE** | 1.78% |
| 3 | **CAN** | 7.99% |  | 24 | **FIN** | 1.56% |
| 4 | **GBR** | 5.59% |  | 25 | **SVN** | 1.51% |
| 5 | **FRA** | 4.18% |  | 26 | **TWN** | 1.41% |
| 6 | **DEU** | 4.12% |  | 27 | **ROW** | 1.36% |
| 7 | **AUS** | 3.99% |  | 28 | **DNK** | 1.31% |
| 8 | **BEL** | 3.46% |  | 29 | **CYP** | 1.26% |
| 9 | **NLD** | 3.23% |  | 30 | **JPN** | 1.02% |
| 10 | **HUN** | 3.21% |  | 31 | **LVA** | 0.83% |
| 11 | **SWE** | 3.17% |  | 32 | **RUS** | 0.78% |
| 12 | **AUT** | 3.11% |  | 33 | **LTU** | 0.72% |
| 13 | **BRA** | 2.95% |  | 34 | **U.S.** | 0.67% |
| 14 | **PRT** | 2.92% |  | 35 | **IND** | 0.66% |
| 15 | **ITA** | 2.75% |  | 36 | **IDN** | 0.65% |
| 16 | **GRC** | 2.38% |  | 37 | **EST** | 0.61% |
| 17 | **TUR** | 2.23% |  | 38 | **KOR** | 0.59% |
| 18 | **ESP** | 2.21% |  | 39 | **ROU** | 0.58% |
| 19 | **POL** | 2.20% |  | 40 | **BGR** | 0.54% |
| 20 | **MEX** | 2.20% |  | 41 | **CHN** | 0.44% |
| 21 | **MLT** | 2.06% |  |  |  |  |

**Supplementary Table 6. US-MNE´ value added, US-MN´ footprint and carbon intensity by sector in developed economies**

|  |  | **US-MNE' carbon footprint** | | **US-MNE' value added** | | **Footprint per unit of value added** |
| --- | --- | --- | --- | --- | --- | --- |
| **SECTOR** | | **GtCO_2_** | **%** | **Million $** | **%** | **GtCO_2_/$** |
| 1 | Mining | 0.0027 | 1% | 33296 | 5% | 0.08 |
| 2 | Food | 0.0209 | 8% | 20407 | 3% | 1.03 |
| 3 | Chemicals | 0.0390 | 15% | 73955 | 10% | 0.53 |
| 4 | Primary and fabricated metals | 0.0026 | 1% | 10544 | 1% | 0.25 |
| 5 | Machinery | 0.0084 | 3% | 22418 | 3% | 0.38 |
| 6 | Computers and electronic products | 0.0094 | 4% | 26736 | 4% | 0.35 |
| 7 | Electrical equipment, appliances, and components | 0.0016 | 1% | 5236 | 1% | 0.31 |
| 8 | Transportation Equipment | 0.0224 | 8% | 31954 | 5% | 0.70 |
| 9 | Rest of Manufacturing | 0.0475 | 18% | 129920 | 18% | 0.37 |
| 10 | Other Industries | 0.0836 | 32% | 63543 | 9% | 1.32 |
| 11 | Wholesale Trade | 0.0113 | 4% | 93261 | 13% | 0.12 |
| 12 | Retail Trade | 0.0060 | 2% | 41823 | 6% | 0.14 |
| 13 | Information | 0.0040 | 2% | 37501 | 5% | 0.11 |
| 14 | Finance and insurance | 0.0040 | 1% | 60968 | 9% | 0.07 |
| 15 | Professional, scientific, and technical services | 0.0017 | 1% | 58124 | 8% | 0.03 |
| 16 | Other Services | 0.0000 | - | 0 | - | - |
| **TOTAL** | | **0.2652** |  | **709686** |  | **0.37** |

**Supplementary Table 7. US-MNE value added, US-MNE´ carbon footprint and carbon intensity by sector in developing economies**

|  |  | **US-MNE' carbon footprint** | | **US-MNE' value added** | | **Footprint per unit of value added** |
| --- | --- | --- | --- | --- | --- | --- |
| **SECTOR** | | **GtCO_2_** | **%** | **Million $** | **%** | **GtCO_2_/$** |
| 1 | Mining | 0.0037 | 2% | 106266 | 27% | 0.03 |
| 2 | Food | 0.0273 | 11% | 11596 | 3% | 2.35 |
| 3 | Chemicals | 0.0331 | 14% | 36622 | 9% | 0.90 |
| 4 | Primary and fabricated metals | 0.0007 | 0% | 2946 | 1% | 0.23 |
| 5 | Machinery | 0.0128 | 5% | 10817 | 3% | 1.18 |
| 6 | Computers and electronic products | 0.0234 | 10% | 23278 | 6% | 1.01 |
| 7 | Electrical equipment, appliances, and components | 0.0050 | 2% | 4418 | 1% | 1.13 |
| 8 | Transportation Equipment | 0.0127 | 5% | 10706 | 3% | 1.19 |
| 9 | Rest of Manufacturing | 0.0632 | 26% | 55750 | 14% | 1.13 |
| 10 | Other Industries | 0.0396 | 16% | 12278 | 3% | 3.23 |
| 11 | Wholesale Trade | 0.0104 | 4% | 53442 | 14% | 0.19 |
| 12 | Retail Trade | 0.0012 | 0% | 4811 | 1% | 0.24 |
| 13 | Information | 0.0033 | 1% | 13690 | 4% | 0.24 |
| 14 | Finance and insurance | 0.0023 | 1% | 22787 | 6% | 0.10 |
| 15 | Professional, scientific, and technical services | 0.0044 | 2% | 20227 | 5% | 0.22 |
| 16 | Other Services | 0.0000 | - | 0 | - | - |
| **TOTAL** | | **0.0.2430** |  | **389634** |  | **0.62** |

**Supplementary Note 1**

One of the main two sources of data is the information about U.S. multinationals activity provided by the Bureau of Economic Analysis (BEA)^1^. This paper uses International Data from BEA, specifically, takes data from the “Data on activities of multinational enterprises” for the investment case of “United States direct investment abroad”. The entity considered is “All majority-owned Foreign Affiliates”, affiliates in which the combined ownership of all U.S. parents exceeds 50 percent. The participation of the affiliates is captured using four indicators of activity abroad: Value Added (Gross Product), Compensation of Employees, Capital Compensation (estimated as Profits plus Capital consumption allowances) and Employment. Data are classified by “Country and Industry” for 2009 for all the 14 industries and 40 countries provided by BEA.

The second main source of data is the MRIO tables and environmental satellite accounts provided by World Input-Output Database (WIOD) in the 2013 release of data^2^, which provides annual time‐series of world input–output tables from 1995 to 2011 but environmental information until 2009. Therefore, we have taken the decision to establish 2009 as our year of study being the last year with complete information about global emissions in this database. Multiregional databases available do not get much further in time, for example, the last release of EXIOBASE (version 3.4) provides information about 2010. Other authors as Meng et al^3^, which analyze the burden of CO_2_ emissions reductions of large Chinese companies in a context of GVC, is based on data for the year 2010. They justify the use of this year for some reasons that can be extrapolated to our analysis such as the stability of the economic structure during the period and the stability of the energy use structure (especially in the period 2009 – 2014).

Regarding countries and regions, WIOD 2013 release covers 27 EU countries and 13 other major countries in the world (Supplementary Figure 1). The remaining non-covered part of the world economy is called “rest of the world” (RoW) region is estimated. Given the relatively large size of this region (with a share of world GDP of around 15%), this region cannot be ignored in analyses of global trade. A detailed description of the estimation process of this region can be found in Timmer et al^2^.

Finally, the WIOD industry disaggregation is 35 sectors, so we have proceeded to aggregate WIOD into the 16 industries of the BEA data for multinationals.

**Supplementary Note 2**

To estimate the $\mathbf{m}_{o}^{c}$ vector we combine two main databases, BEA’s AMNE database and WIOD. This combination is similar to those followed by ^4^ (that combine OECD AMNE and WIOD) and by ^5^ (that combine BEA’s AMNE and U.S. SUT), although with different final goals and procedures. The combination of the two sources can provide some biases in the results at sector level (not in total country figures) related to the different unit considered, establishment in WIOD and enterprise in AMNE. The differences are reduced to the presence of several affiliates with activity in different sectors because the BEA explicitly identifies the primary industry of the controlling unit, as well as the primary industry of its affiliates, according to the type of products with the highest share in sales^6^. The calculations are as follows. First, we extract the US-MNE activity (value added, for instance) from BEA for each sector in each country (section ‘Activities of U.S. Multinational Enterprises (MNEs)’). Second, we divide it by the total value added (or the appropriate indicator) of each sector in each country taken from WIOD to obtain the share of value added generated by the US-MNE affiliate activity. Although the main estimations of this paper are based on the value added (gross product) generated abroad by US-MNE affiliates, three other alternative indicators of US-MNE activity abroad have been taken into account: Compensation of Employees, Capital Compensation and Employment, following analogous calculations. Finally, four different $\mathbf{m}_{o}^{c}$ are estimated, one for each indicator of US-MNE activity. The $\mathbf{m}_{o}^{c}$ vectors are shown in Supplementary Data 8 to 11.

**Supplementary References**

1. BEA. International Data: Direct Investment and MNE. (ed^(eds). Bureau of Economics Analysis (BEA). U.S. Department of Commerce (Various years).

2. Timmer MP, Dietzenbacher E, Los B, Stehrer R, de Vries GJ. An Illustrated User Guide to the World Input–Output Database: the Case of Global Automotive Production. *Review of International Economics* **23**, 575-605 (2015).

3. Meng B*, et al.* More than half of China’s CO2 emissions are from micro, small and medium-sized enterprises. *Applied Energy* **230**, 712-725 (2018).

4. Cadestin C, Backer KD, Desnoyers-James I, Miroudot S, Rigo D, Ye M. Multinational enterprises and global value chains: the OECD analytical AMNE database. (2018).

5. Fetzer JJ, Highfill T, Hossiso KW, Howells TF, III, Strassner EH, Young JA. Accounting for Firm Heterogeneity within U.S. Industries: Extended Supply-Use Tables and Trade in Value Added using Enterprise and Establishment Level Data. *National Bureau of Economic Research Working Paper Series* **No. 25249**, (2018).

6. BEA. U.S. International Economic Accounts: Concepts and Methods. (ed^(eds). Bureau of Economic Analysis, U.S. Department of Commerce. (2014).
