# Supplementary material for: The carbon footprint of the U.S. multinationals’ foreign affiliates
Source: Nat Commun. 2019 Apr 11;10:1672. doi: 10.1038/s41467-019-09473-7 (PMC6459871; doi:10.1038/s41467-019-09473-7)
Supplement: Supplementary file 3 — Description of Additional Supplementary Files [file 41467_2019_9473_MOESM3_ESM.pdf]

Description of Additional Supplementary Info

File Name: Supplementary Data 1
Description: Table of data of Figure1. 2009 US-MNE carbon footprint among the world ranking of emitters (GtCO2).

File Name: Supplementary Data 2
Description: Table of data of Figure 2. US-MNE carbon footprint by host country, the producer footprint (GtCO2).

File Name: Supplementary Data 3
Description: Table of data of Figure 3. 2009 weight of US-MNEs' carbon footprint on producer responsibility of the host country (producer footprint).

File Name: Supplementary Data 4
Description: Table of data of Figure 4. 2009 US-MNEs' carbon footprint by Consumer Country (MNE CF) (GtCO2).

File Name: Supplementary Data 5
Description: Table of data of Figure 5. Carbon flows embodied in US-MNEs' exports operating abroad.

File Name: Supplementary Data 6
Description: Table of data of Figure 6. Shares of US-MNEs' value added (left) and producer footprint (right) by sectors (%).

File Name: Supplementary Data 7
Description: Table of data of Figure 7. 2009 US-MNEs' carbon footprint in developed and developing countries by indicator and scenario (GtCO2).

File Name: Supplementary Data 8
Description: US-MNE' value added participation over total value added.

File Name: Supplementary Data 9
Description: US-MNE' compensation of employees participation over total compensation of employees.

File Name: Supplementary Data 10
Description: US-MNE' employment participation over total employment.

File Name: Supplementary Data 11
Description: US-MNE' capital compensation participation over total capital compensation.

File Name: Supplementary Data 12
Description: Data on activities of multinational enterprises.

File Name: Supplementary Data 13
Description: Data on activities of multinational enterprises.

File Name: Supplementary Data 14
Description: Data on activities of multinational enterprises.

File Name: Supplementary Data 15
Description: Data on activities of multinational enterprises.

File Name: Supplementary Data 16
Description: Code used to perform the MRIO analysis.
